# Hinokitiol up-regulates miR-494-3p to suppress BMI1 expression and inhibits self-renewal of breast cancer stem/progenitor cells

**DOI:** 10.18632/oncotarget.18648

**Published:** 2017-06-27

**Authors:** Shih-Ming Chen, Bing-Yen Wang, Che-Hsin Lee, Hsueh-Te Lee, Jung-Jung Li, Guan-Ci Hong, Yu-Chieh Hung, Peng-Ju Chien, Che-Ying Chang, Li-Sung Hsu, Wen-Wei Chang

**Affiliations:** ^1^ Institute of Biochemistry, Microbiology and Immunology, Chung Shan Medical University, Taichung, Taiwan; ^2^ Division of Thoracic Surgery, Department of Surgery, Changhua Christian Hospital, Changhua City, Taiwan; ^3^ School of Medicine, Chung Shan Medical University, Taichung, Taiwan; ^4^ School of Medicine, College of Medicine, Kaohsiung Medical University, Kaohsiung, Taiwan; ^5^ Institute of Genomics and Bioinformatics, National Chung Hsing University, Taichung, Taiwan; ^6^ Department of Biological Sciences, National Sun Yat-sen University, Kaohsiung, Taiwan; ^7^ Institute of Anatomy and Cell Biology, School of Medicine, National Yang Ming University, Taipei City, Taiwan; ^8^ Department of Biomedical Sciences, Chung Shan Medical University, Taichung, Taiwan; ^9^ Department of Medical Research, Chung Shan Medical University Hospital, Taichung, Taiwan

**Keywords:** hinokitiol, miR-494-3p, BMI1, breast cancer, cancer stem cells

## Abstract

Hinokitiol (β-thujaplicin) is a tropolone-related compound that has anti-microbe, anti-inflammation, and anti-tumor effects. Cancer stem/progenitor cells (CSCs) are a subpopulation of cancer cells with tumor initiation, chemoresistant, and metastatic properties and have been considered the important therapeutic target in future cancer therapy. Previous studies reported that hinokitiol exhibits an anti-cancer activity against murine tumor cells through the induction of autophagy. The current research revealed that hinokitiol suppressed the self-renewal capabilities of human breast CSCs (BCSCs) and inhibited the expression of BMI1 at protein level without suppressing its mRNA. Treatment of hinokitiol in mammospheres induced the expression of miR-494-3p and inhibition of miR-494-3p expression in BCSCs. This treatment abolished the suppressive effects of hinokitiol in mammosphere formation and BMI1 expression. BMI1 is a target of miR-494-3p by luciferase-based 3′UTR reporter assay. Overexpression of miR-494-3p in BCSCs caused the down-regulation of BMI1 protein, inhibition of mammosphere forming capability, and suppression of their tumorigenicity. Moreover, miR-494-3p expression was significantly and inversely correlated with patient survival in two independent public database sets. Furthermore, treatment of hinokitiol *in vivo* suppressed the growth of xenograft human breast tumors as well as the expression of BMI1 and ALDH1A1 in xenograft tumors. In conclusion, these data suggest that hinokitiol targets BCSCs through the miR-494-3p-mediated down-modulation of BMI1 expression.

## INTRODUCTION

Cancer stem/progenitor cells (CSCs) are a subpopulation of cancer cells involved in tumor initiation, resistance to treatment, and metastasis [1–3]. These cancer cells are considered as the most important target in the development of cancer therapy [4, 5]. In breast cancer, breast CSCs (BCSCs) have been identified as cells with surface markers of CD24–CD44+ [6] or high intracellular aldehyde dehydrogenase (ALDH) activity [7]. CSC activity could also be determined by tumorsphere cultivation, a non-adherent culture condition that enables the enrichment of CSC population from cancer cell lines or primary cancer cells [8–10]. The polycomb complex protein BMI1 regulates the self-renewal capability of normal and malignant mammary stem cells [11]. Overexpression of BMI1 in normal mammary epithelial cells increased mammosphere formation [11]. Co-overexpression of Bmi1 and activated H-Ras (RasG12V) in MCF10A cells resulted in the formation of poorly differentiated carcinomas with epithelial–mesenchymal transition features in severe combined immunodeficient mice [12]. Recently, a small molecule inhibitor of BMI1, PTC-209, has been identified to cause a decrease in BCSCs through the up-regulation of tumor suppressor microRNAs (miRNAs), such as miR-200 and miR-141 [13].

MiRNAs belong to a family of small non-coding RNAs with 19–24 nucleotides in length. This group functions as gene regulators to suppress gene expression by binding to the 3′-untranslated region to cause translation inhibition or mRNA degradation of their target genes [14]. The functions of miR-494-3p in cancer are controversial. miR-494-3p has been reported to demonstrate oncogenic effects by modulating NOTCH1 and PTEN/PI3K/AKT signaling in non-small cell lung cancer [15], accelerating cell proliferation in liver cancer, and down-regulation or mutation in colorectal cancer [16]. On the other hand, miR-494-3p could inhibit gastrointestinal stromal tumor cell proliferation by targeting KIT [17] or suppressing invasion of prostate cancer cells through down-regulation of CXCR4 [18]. Recently, miR-494-3p was demonstrated to target PAK1 in breast cancer cells, leading to the suppression of cell invasion [19]. However, the role of miR-494-3p in regulation of self-renewal of BCSCs remains unclear.

Hinokitiol is a natural monoterpenoid originally extracted from Taiwanese hinoki and has anti-inflammatory and anti-microbial abilities [20, 21]. Furthermore, hinokitiol induces apoptosis in cancer cells through a caspase 3-dependent pathway or through cell-cycle arrest [22–24]. Previous studies demonstrated that hinokitiol caused cell death in murine breast and colorectal cancer cells through the induction of autophagy [25]. These studies indicated that hinokitiol could serve as a novel anti-cancer compound. The CSC targeting effect of this compound needs further investigation.

The present study reveals that hinokitiol inhibited the self-renewal of BCSCs and down-regulated BMI1 protein expression without affecting its mRNA level. The expression of miR-494-3p in mammospheres was induced by hinokitiol. Inhibition of miR-494-3p abolished the inhibitory effect of hinokitiol in targeting BMI1. Overexpression of miR-494-3p in human breast cancer cells suppressed tumor growth of BCSCs *in vivo*. The expression of miR-494-3p was significantly and inversely correlated with breast cancer patient survival in two independent public database sets. Finally, hinokitiol suppressed tumor growth in a xenograft breast cancer model. The upregulation of miR-494-3p as well as down-regulation of BMI1 was observed in hinokitiol-treated xenograft tumors. In conclusion, hinokitiol could target BCSCs *in vitro* and *in vivo* through upregulation of miR-494-3p, inhibiting BMI1 expression.

## RESULTS

### Hinokitiol's ability to inhibit the self-renewal capabilities of BCSCs

**Figure 1:**
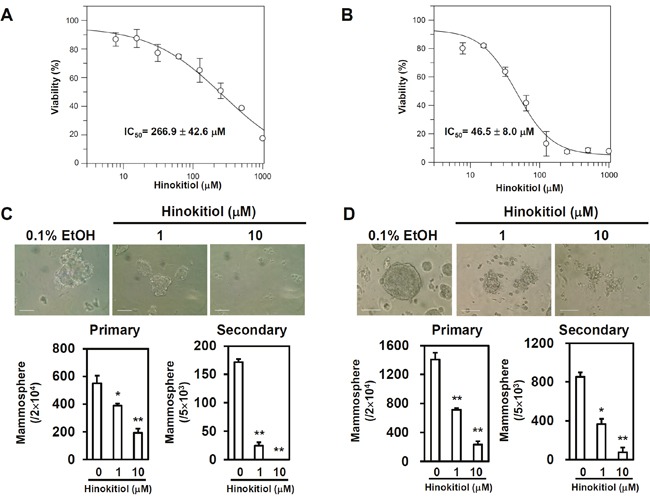
Hinokitiol inhibits self-renewal of BCSCs **(A, B)** The **(A)** AS-B145 and **(B)** BT-474 human breast cancer cells were treated with various concentrations of hinokitiol for 72 h and the cell viability was determined by WST1. IC_50_ values were calculated by GraFit software. **(C, D)** Self-renewal capability of AS-B145 **(C)** or BT-474 **(D)** was determined by mammosphere cultivation. 2×10^4^ cells were used for primary mammosphere formation assay and 5×10^3^ dissociated primary mammosphere cells were used for secondary mammosphere formation assay. Hinokitiol was treated for 7 days. Scale bar: 100 μm. *, p< 0.05; **, p< 0.01. The experiments were repeated at least two times and data from one experiment were presented.

The cytotoxic effects of hinokitiol were examined in two human breast cancer cell lines, AS-B145 and BT-474. As shown in Figure 1, the half-maximal inhibitory concentration (IC_50_) values of hinokitiol in AS-B145 (Figure 1A) and BT-474 (Figure 1B) cells were 266.9 ± 42.6 μM and 46.5 ± 8.0 μM, respectively. The potential anti-CSC effects of hinokitiol was further evaluated in the range of concentrations without causing massive cell death at 0–10 μM with a mammosphere cultivation assay. This assay determines the self-renewal capability of BCSCs [8, 26]. Hinokitiol significantly inhibited primary and secondary mammosphere formation at 10 μM, a concentration below IC_50_ value, in both AS-B145 (Figure 1C) and BT-474 (Figure 1D). These results indicate that hinokitiol displays an anti-self-renewal activity of BCSCs *in vitro*.

### The mediation of miR-494-3p on the suppressive effects of hinokitiol to the self-renewal capability and BMI1 expression in BCSCs

**Figure 2:**
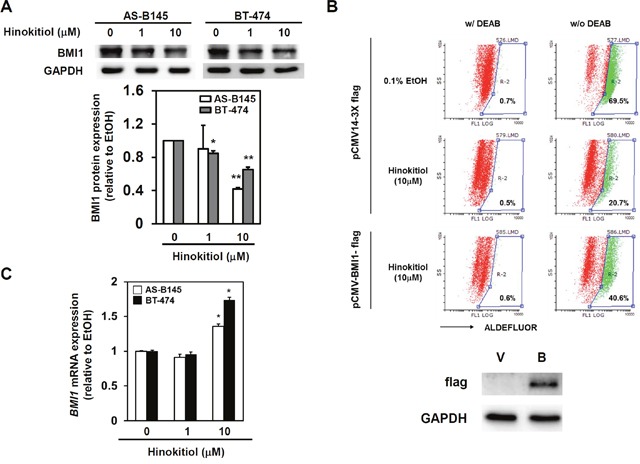
Hinokitiol inhibits BMI1 protein expression, but not mRNA, in BCSCs **(A)** AS-B145 or BT-474 cells were cultured into primary mammospheres and dissociated into single cell suspension by HyQTase treatment. Secondary mammosphere cells were then treated with hinokitiol as 1 or 10 μM for 48 h and harvested for analyzing BMI1 protein expression by western blot. BMI1 protein expression levels were normalized to GAPDH and compared with 0.1% EtOH treated group. *, *P*<0.05; **, *P*<0.01. **(B)** BT-474 cells were transfected with pCMV14-3X flag or pCMV-BMI1-flag for 48 hours and performed mammosphere cultivation under 0.1% ethanol (EtOH) or 10 μM hinokitiol treatment. The ALDH+ BCSCs were determined at Day 7 post treatment by ALDEFLUOR assay and FACS analysis. DEAB (N,N-diethylaminobenzaldehyde) was used for gating ALDH+ population of cells. V, pCMV14-3X flag; B, pCMV-BMI1-flag. **(C)** *BMI1* mRNA expression in hinokitiol treated mammopsheres derived from AS-B145 or BT-474 cells was determined by SYBR Green based qRT-PCR. Data were expressed as the mean ± SD of two independent experiments.

**Figure 3:**
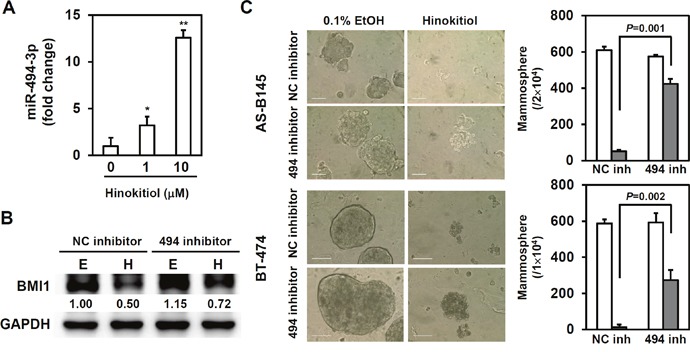
miR-494-3p mediates the suppressive effect of hinokitiol in the self-renewal of BCSCs **(A)** miR-494-3p expression in mammospheres derived from AS-B145 cells at Day 6 post hinokitiol treatment were determined by qRT-PCR. *, *P*<0.05; **, *P*<0.01. **(B)** AS-B145 mammosphere cells were transfected with 100nM of negative control inhibitor (NC inh) or miR-494-3p inhibitor (494 inh) for 24 hours and treated with 0.1% EtOH or 10 μM hinokitiol for further 48 hours. Cells were harvested for determination of BMI1 expression by western blot. **(C)** AS-B145 or BT-474 cells were firstly cultured into primary mammospheres, dissociated into single cell suspension, transfected with NC inh or 494 inh for 24 hours and performed secondary mammosphere cultivation under the treatment of 0.1% EtOH or 10 μM hinokitiol. Secondary mammosphere number was counted at Day 7 and data were expressed as the mean ± SD of triplicate determinations. White bar, EtOH treated group; gray bar, hinokitiol treated group. Scale bar= 100 μm. The experiments were repeated at least two times and data from one experiment were presented.

**Figure 4:**
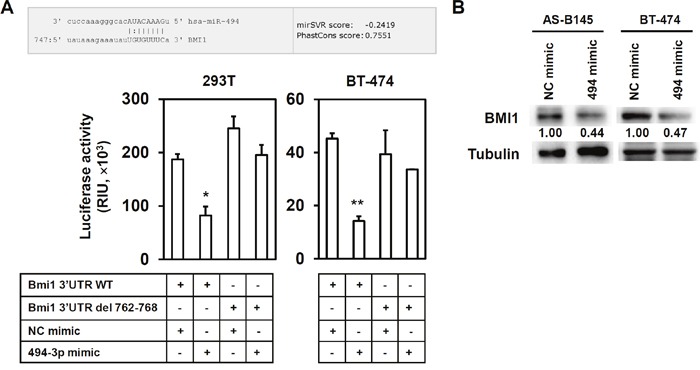
BMI1 is a target of miR-494-3p **(A)** The alignment of BMI1 3’-UTR and miR-494-3p was obtained from the website of MICRORNA. ORG (http://www.microrna.org). 293-T or AS-B145 cells were transfected with a negative control (NC) mimic or miR-494-3p (494-3p) mimic at a concentration of 100 nM together with wildtype BMI1 3’-UTR (BMI1 3’-UTR WT) or mutant from (BMI1 3’UTR del 762-768) for 48 h and determined luciferase activities. Data were presented as mean±SD. *, *P*<0.05; **, *P*<0.01. **(B)** AS-B145 or BT-474 cells were firstly cultured into primary mammospheres, dissociated into single cell suspension and transfected with NC or 494-3p mimic at a concentration of 100 nM for 48 hours. BMI1 expression was then determined by western blot. Inset values indicate protein expression normalized to tubulin. The experiments were repeated at least two times and data from one experiment were presented.

BMI1 positively regulates the self-renewal capability of BCSCs [11, 12]. The effect of hinokitiol to the BMI1 expression of mammospheres in AS-B145 and BT-474 breast cancer cells was examined. As shown in Figure 2, hinokitiol significantly inhibited BMI1 expression in both AS-B145- and BT-474-derived mammospheres at a concentration of 10 μM (Figure 2A). BMI1 was further overexpressed in BT-474 cells in order to reduce the therapeutic effects of hinokitiol in targeting BCSCs. The ALDEFLUOR assay findings show that hinokitiol treatment obviously decreased ALDH+ BCSCs within BT-474 mammospheres (from 69.5% to 20.7%), but the inhibition was less efficient in BMI1-overexpressing cells (the ALDH+ cells remained 40.6%) (Figure 2B). However, hinokitiol did not suppress the mRNA expression of BMI1 in AS-B145- or BT-474-derived mammospheres (Figure 2C). This result suggests that the inhibitory effects of hinitiol to BMI1 expression may be mediated by a specific miRNA. Previous experiments demonstrated that BMI1 was a target of miR-494-3p in oral squamous carcinoma cells [27]. The expression of miR-494-3p in mammospheres was then detected after hinokitiol treatment. Results showed that hinikitiol induced miR-494-3p in mammospheres derived from AS-B145 cells (Figure 3A). When AS-B145 or BT-474 cells were transfected with miR-494-3p inhibitor, the inhibitory effects of hinokitiol to BMI1 expression (Figure 3B) or mammosphere formation (Figure 3C) was abolished. The results of the luciferase reporter assay suggest that transfection of miR-494-3p mimic into 293T human embryonic kidney cells or BT-474 breast cancer cells significantly reduced the activity of luciferase fused with wildtype 3′-UTR of BMI1, but not to the luciferase fused with mutated 3′-UTR by deletion of miR-494-3p binding sites (Figure 4A). Furthermore, overexpression of miR-494-3p in AS-B145 and BT-474 mammospheres suppressed BMI1 expression (Figure 4B). These results indicate that the inhibitory effect of hinokitiol to the self-renewal capability of BCSCs is mediated by miR-494-3p-induced BMI1 down-regulation.

### miR-494-3p as an oncosuppressor miRNA in breast cancer

**Figure 5:**
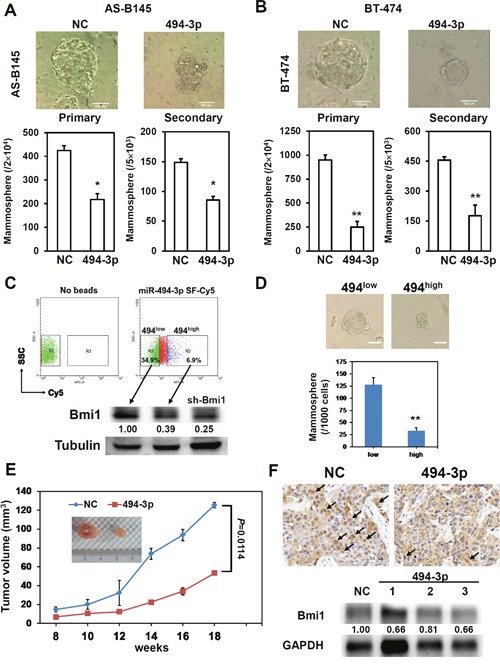
miR-494-3p serves as an oncosuppressor in breast cancer **(A, B)** The AS-B145 **(A)** or BT-474 **(B)** cells were transfected with negative control (NC) or miR-494-3p (494-3p) mimic at a concentrations of 100 nM for 24 hours and performed primary mammosphere cultivation. The number of formed primary mammospheres was counted at Day 7 and collected for second time transfection with NC or 494-3p mimic. After transfection for 24 hours, the cells were used for secondary mammosphere cultivation and counted the formed mammospheres at Day 7. *, *P*<0.05; **, *P*<0.01. Scale bar= 100 μm. **(C, D)** BT-474 cells were incubated with miR-494-3p Smartflare beads for 16 hours and sorted into miR-494-3p^low^ (494^low^, the fluorescence intensity lower than 10 as similar to no beads control) or miR-494-3p^high^ (494^high^, the fluorescence intensity higher than 30) cells and detected the BMI1 expression by western blot **(C)**. sh-Bmi1 transduced mammosphere cells from BT-474 were used as a control. The sorted 494^low^ or 494^high^ cells were then performed mammosphere cultivation and the number of formed mammosphere was pictured and counted at Day 7. **, p< 0.01. Scale bar= 50 μm. **(E, F)** BT-474 cells were transfected with NC or 494-3p mimic at a concentration of 100 nM for 24 hours and cells were harvested for xenograftment assay by injection into mammary fads of NOD/SCID mice **(E)**. The formed tumors were taken and analyzed BMI1 expression by immunohistochemistry or western blot **(F)**. Arrows indicated tumor cells with nuclear BMI1 expression.

**Figure 6:**
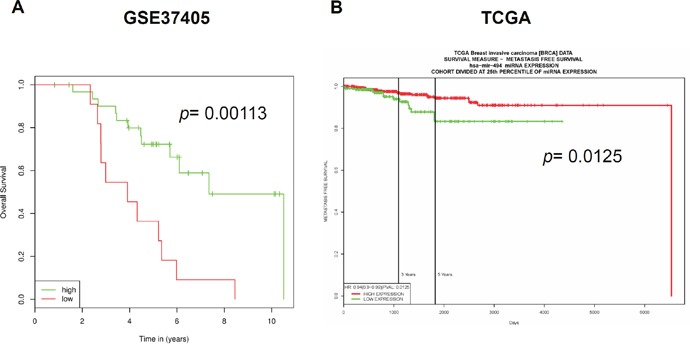
The expression of miR-494 is negatively correlated with survival time of breast cancer patients The correlation between miR-494 expression and overall survival in a GSE37405 dataset of ER+ breast cancer patients **(A)** or metastasis free survival in TCGA invasive breast cancer dataset **(B)** was analyzed by MIRUMIR or PROGmiR V2 website.

With transfection of miR-494-3p mimic, the overexpression of miR-494-3p significantly suppressed the primary and secondary mammosphere formations of AS-B145 (Figure 5A) and BT-474 cells (Figure 5B). FACS was performed to analyze the intracellular miRNA expression utilizing Smartflare fluorescent beads [28]. In this analysis, BT-474 cells were further sorted into two populations of miR-494-3p^low^ and miR-494-3p^high^ groups with Smartflare miR-494-3p fluorescent probe (Figure 5C). Results show that the expression of BMI1 protein was higher in miR-494-3p^low^ BT-474 cells than those of miR-494-3p^high^ counterparts (Figure 5C). With mammosphere assay, the miR-494-3p^high^ BT-474 cells displayed a poor CSC activity that formed less number of mammospheres, as well as decreased in mammosphere size, than the miR-494-3p^low^ counterparts (Figure 5D). The miR-494-3p overexpression was introduced by the lentiviral delivery of miR-494-3p precursor into the BT-474 cells and performed xenograftment assay *in vivo* in NOD/SCID immunocompetent mice; the tumor growth of BT-474 cells with miR-494-3p overexpression was significantly slower than control tumors (Figure 5E, *p*= 0.0114). Immunohistochemistry analysis demonstrated a reduction of the positive stain of nuclear BMI1 in tumors derived from miR-494-3p overexpressed BT-474 cells (Figure 5F). Furthermore, the down-regulation of BMI1 protein expression in xenograft tumors derived from miR-494-3p overexpressed BT-474 cells was confirmed by Western blot (Figure 5F). Kaplan–Meier survival analysis was performed in breast cancer patients from GSE37405 dataset (overall survival among ER+ breast cancer patients) by MIRUMIR (Figure 6A) and the Cancer Genome Atlas (TCGA) dataset (metastasis-free survival among invasive breast carcinoma patients) by PROGmiR V2 online tools (Figure 6B). The results showed that the lower expression of miR-494 had a significant poor survival time (Figure 6, *p*= 0.00113 for GSE37405 and *p*=0.0125 for TCGA dataset). Moreover, the results indicate that miR-494-3p functions as an oncosuppressor in breast cancer.

### The anti-breast cancer effect of hinokitiol *in vivo* associated with the down-regulation of BMI1

**Figure 7:**
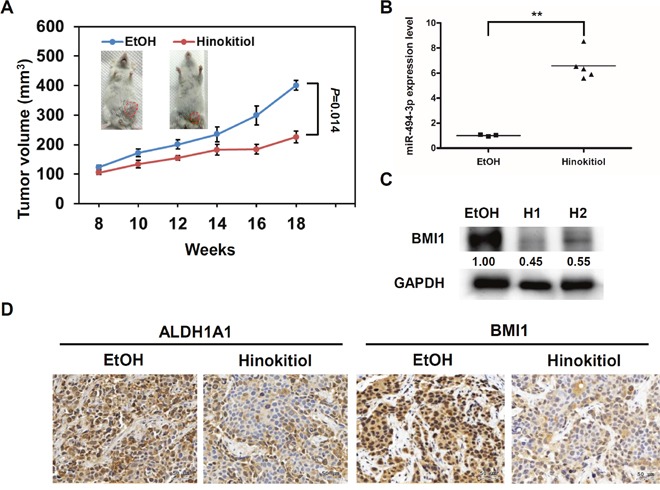
Hinokitiol reduces *in vivo* tumorigenicity of BCSCs BT-474 cells were firstly cultured into mammospheres and 1×10^5^ cells were injected into mammary fat pads of NOD/SCID mice for tumor growth. **(A)** The treatment of hinokitiol at a dose of 40mg/kg was performed when tumors reached 100 mm^3^ by twice/week until 18 weeks. **(B)** miR-494-3p expression in each formed tumor was determined by qRT-PCR. **, *P*< 0.01. **(C)** BMI1 expression in tumors was determined by western blot. H1 or H2 represented independent tumor samples from hinokitiol treated mice. GAPDH was used as protein loading control. The inserted numbers indicated the relative expression level of BMI1 when compared to the EtOH treated sample. **(D)** The expression of ALDH1A1 and BMI1 in formed tumors was determined by immunohistochemistry.

To examine the therapeutic potential of hinokitiol *in vivo* to breast cancer, the BT-474 mammosphere cells were injected into the mammary fat pads of NOD/SCID and treated with 40 mg/kg hinokitiol intraperitoneally when the tumor volume reached to 100 mm^3^. Compared with ethanol-treated group, the tumor growth of hinokitiol-treated group was significantly reduced (Figure 7A, *p*=0.014). By qRT-PCR analysis, the expression levels of miR-494-3p in hinokitiol-treated tumors were significantly increased when compared to ethanol-treated group (Figure 7B, *p*=0.0002). The BMI1 protein expression level in hinokitiol-treated tumor samples was reduced as compared with the EtOH-treated tumor in Western blot analysis (Figure 7C). By immunohistochemistry analysis, the expression level of ALDH1A1, one of the BCSC markers [7], and nuclear BMI1 was decreased in hinokitiol-treated tumors (Figure 7D). These data suggest that the therapeutic effect of hinokitiol *in vivo* in the suppression of breast tumor growth is associated with the upregulation of miR-494-3p, leading to the down-regulation of BMI1 expression.

## DISCUSSION

Hinokitiol displays an anti-cancer activity by the induction of cell-cycle arrest [23], apoptosis [24], DNA damage [29], or autophagic cell death [25]. This study reports that the anti-CSC activity of hinokitiol was partially mediated by miR-494-3p. The inhibition of miR-494-3p expression partially abolished the anti-BCSC effects of hinokitiol (Figure 3). This experiment shows that the anti-cancer activity of hinokitiol is mediated by the induction of an oncosuppressor miRNA. Hibino et al. have demonstrated that inhibition of the enhancer of zeste homolog 2 (EZH2) by 3-deazaneplanocin A induced tumor suppressor miRNAs in liver cancer cells [30]. A similar phenomenon was also found in a recent multiple myeloma study [31]. In hinokitiol-treated xenograft tumors derived from BT-474 mammospheres, the down-regulation of EZH2 was observed when compared with EtOH-treated group (Supplementary Figure 1). This finding suggests that hinokitiol may induce miR-494-3p expression through the down-regulation of EZH2, but the underlying molecular mechanism requires further investigation. Previous experiments reveal that hinokitiol could induce proteasomal degradation of epidermal growth factor receptor (EGFR), which led to the suppression of vasculogenic mimicry activity of BCSCs [32]. The EGFR signaling has been demonstrated to contribute to the enhancement of BCSC self-renewal induced by versican [33] or by tumor-associated macrophages [34]. Downregulation of EGFR expression by hinokitiol may also involve in the anti-BCSC effect but remains to be further investigated. The relationship between the expression of miR-494-3p and EGFR in breast cancer cells will be further investigated.

The function of miR-494-3p in carcinogenesis is controversial. Several reports suggested that miR-494-3p is an oncogenic miRNA. This miRNA shortens the disease-free survival time in lung cancer patients with high expression levels [15] or the inhibition of PTEN by miR-494-3p, leading to the activation of Akt in nasal natural killer cell lymphoma [35]. By contrast, other reports indicated that miR-494-3p could be a tumor suppressor miRNA. The direct targeting of Sox9 by miR-494-3p led to the suppression of cell migration, invasion, as well as tumor growth of chondrosarcoma cells [36]. The dual role of a single miRNA in carcinogenesis would not be surprising. miR-375 functioned as both an oncomiR and tumor suppressor miRNA in prostate cancer, depending on the stage of tumor progression and hormone status [37]. Furthermore, the effect of miRNAs to carcinogenesis mainly depends on the cellular context in different tumors [38]. This study presents the tumor-suppressive function of miR-494-3p in breast cancer by inhibiting BCSC self-renewal and directly targeting BMI1 (Figure 4 and 5). These findings are consistent with the previous studies, which demonstrated that miR-494-3p induced cellular senescence in oral squamous carcinoma cells through the downregulation of BMI1 [27]. These results also show the tumor suppression effects of miR-494-3p. In addition, miR-494-3p was negatively correlated with the overall survival of ER+ breast cancer patients (GSE37405) (Figure 6A) or metastasis-free survival of breast invasive carcinoma patients (TCGA data) (Figure 6B). These data were consistent with a recent study from Zhan et al., showing that the expression of miR-494 was significantly reduced in tumors when compared with adjacent non-neoplastic breast tissues [19]. Altogether, these data strongly suggest that miR-494-3p is an oncosuppressor miRNA in breast cancer.

The current research shows that irradiation could induce epithelial–mesenchymal transition in breast cancer cells by the upregulation of BMI1 [39]. Knockdown of BMI1 abolished irradiation-induced cell migration in breast cancer cells [39]. Results regarding the inhibitory effect of hinokitiol in BMI1 expression through the induction of miR-494-3p suggest that hinokitiol has the potential to develop into a sensitization agent in breast cancer radiotherapy. On the other hand, utilization of miR-494-3p oligos as an enhancer is also recommended to facilitate the therapeutic efficiency of hinokitiol in the future. In conclusion, these data demonstrated that hinokitiol could suppress the self-renewal and tumorigenicity of BCSCs through miR-494-3p-mediated BMI1 inhibition. These findings suggest that hinokitiol has a potential to be developed as a chemoprevention agent in breast cancer.

## MATERIALS AND METHODS

### Cell lines and reagents

AS-B145 cells were cultured in Minimum Essential Medium (MEM) Alpha Medium supplemented with 2 mM L-glutamine, 10% heat-inactivated fetal bovine serum and 5 μg/ml insulin at 37°C in 5% CO_2_. BT-474 cells were cultured in Dulbecco's modified Eagle medium and Ham's F-12 (DMEM/F12) medium (1:1) supplemented with 2 mM L-glutamine and 10% heat-inactivated fetal bovine serum (FBS) at 37°C in 5% CO_2_. Hinokitiol was purchased from Sigma-Aldrich (Sigma-Aldrich, St. Louis, MO) and dissolved in absolute ethanol as a stock of 100 mM.

### Cell viability assay

Cells were treated with a sequential concentration of hinokitiol (0-1000 μM) and cultured for 48 h. Cell survival was assessed using WST-1 reagent by measuring absorbance at 440nm wavelength. The half maximal inhibitory concentration (IC50) value was calculated by GraFit software (version 7, Erithacus Software Ltd., Surrey, UK)

### Immunoblot analysis

The total protein concentration in each sample was determined with the bicinchoninic acid (BCA) protein assay (Pierce Biotechnology, Rockford, IL, USA). Proteins were separated by SDS-PAGE, transferred onto Polyvinylidene Fluoride (PVDF) membranes (Pall Corporation, Port Washington, NY, USA), and probed with rabbit polycolnal anti-BMI1 (Novus Biologicals, Littleton, CO, USA) or rabbit polyclonal anti-GAPDH (GeneTex International Corporation, Hsinchu City, Taiwan). Horseradish peroxidase-conjugated goat anti-rabbit IgG polyclonal antibody (GeneTex) was used as the secondary antibody followed by incubation with T-Pro LumiFast Chemiluminescent Substrate (JF Ji-Feng Biotechnology, New Taipei City, Taiwan) and the signals were visualized and captured with a FUSION Solo S Imaging system (Vilber Lourmat, MArne-la-Valée, France). The signals were quantified with ImageJ software (version 1.51i, National Institute of Mental Health, Bethesda, MA, USA). For quantification of BMI1 protein in xenograft tumors by western bot, 10 μm of paraffin embedded tumors were sliced and proteins were extracted according to the protocol of Guo et al. [40].

### Mammosphere cultivation

Mammopshere cultivation was performed as previously described [41, 42]. Briefly, Cells were suspended into DMEM/F12 medium supplemented with 0.4% bovine serum albumin (Sigma-Aldrich), 10 ng/ml epidermal growth factor (PeproTech Asia, Rehovot, Israel), 10 ng/ml basic fibroblast growth factor (Sino Biological Inc., Beijing, China), 0.5X B27 supplement (Thermo Fisher Scientific, Waltham, MA, USA), 5 μg/ml insulin (Sigma-Aldrich), 1 μg/ml hydrocortisone (Sigma-Aldrich) and 4 μg/ml heparin (Sigma-Aldrich) and seeded into ultralow attachment 6-well-plate (Greiner Bio-One GmbH, Kremsmünster, Austria) at a density of 1×10^4^/ml. The number of formed primary mammospheres with a diameter larger than 50 μm was counted under an inverted microscopy. Primary mammospheres were harvested by filtering with 70 μm cell strainer (BD Biosciences), dissociated into single cell suspension by HyQTase and performed secondary mammosphere formation as the protocol of primary mammosphere cultivation.

### Quantitative reverse transcription-polymerase chain reaction (qRT-PCR)

qRT-PCR was performed as previously described [27]. Briefly, total RNA was extracted and purified by Quick-RNA™ MiniPrep Plus (Zymo Research Corp, Irvine, CA, USA). 1 μg total RNA was used for complementary DNA (cDNA) conversion by RevertAid First Strand cDNA Synthesis Kit (Thermo Fisher Scientific). For miR-494-3p detection, a specific RT primer for miR-494-3p (Guangzhou RiboBio Co., Ltd., Guangzhou, China) was used for cDNA conversion and qPCR was then performed by KAPA SYBR^®^ FAST qPCR Kit (Kapa Biosystems, Inc., Wilmington, MA, USA) and StepOnePlus™ Real-Time PCR System (Thermo Fisher Scientific) with specific qPCR primer pair (Guangzhou RiboBio Co., Ltd.) under a condition as described in [27]. RNU6B was used as internal control for analyzing miR-494-3p expression. The primers sequences for detection of *BMI1* and *MRPL19* were used as described in [27].

### Transfection

The transfection of plasmid DNA, miRNA mimic or inhibitor was performed by TurboFect Transfection Reagent (Thermo Fisher Scientific) with the manufacture's protocol. Briefly, 100 nM miRNA mimic or inhibitor (purchased from Guangzhou RiboBio Co., Ltd.) or 1 μg of pCMV14-3X flag or pCMV-BMI1-flag was complexed with transfection reagent as a ratio of 1 μg nuclei acid: 2 μl reagent at room temperature for 15 minutes and then added into wells of 6-well-plates with cell attachment at 60% confluency. Cells were then harvested at 48h post transfection for further experiments.

### Constructs and luciferase-based reporter assay

Human *BMI1* gene was amplified by PCR from cDNA of BT-474 cells and cloned into pCMV14-3X flag vector with following primers: KpnI-BMI1-F (5'-CgCggTAccATgCATCgAACAACgAgAATC-3') and BMI1ns-BamHI-R (5'-AgCggATCCACCAgAAgAAgTTgCTgATgAC-3'). The firefly luciferase reporter plasmid with full length *BMI1* 3’-UTR was purchased from OriGene Technologies, Inc. (Rockville, MD, USA) and a reporter plasmid of mutant *BMI1* 3’-UTR with a deletion of putative miR-494-3p binding region (deletion of nucleotide positions from 762 to 768 in 3’-UTR of *BMI1*) was constructed with site-directed mutagenesis kit as described in [27]. For reporter assay, the firefly luciferase reporter plasmid was mixed with a renilla luciferase plasmid as a ratio of 50:1 and transfected together with miR-494-3p mimic or negative control mimic into 293T or BT-474 cells. The luciferase activity was detected at 48h post transfection as described in [27].

### Fluorescence-activated cell sorting (FACS)

ALDH+ BCSCs within BT-474 mammospheres were detected by ALDEFLUOR assay (StemCell Technologies, Inc., Vancouver, BC, Canada) according to our previous report [43]. The fluorescence signals were analyzed by COULTER^TM^ Epics XL flow cytometry (Beckman Coulter, Inc. Brea, CA, USA). In order to sort the differential miR-494-3p expressing cells, the Smartflare beads was used (Merck Millipore, Temecula, CA, USA). BT-474 cells were grew in a 10 cm dish for 80% confluency with 8 ml culture medium, added 8 μl miR-494-3p Smartflare-Cy5 beads and then incubated at 37°C for 16 hours. After incubation, cells were harvested with trypsin/EDTA and suspended in DMEM/F12 supplemented with 5% FBS and performed cell sorting with FACSAria cell sorter (BD Biosciences).

### Human breast cancer xenograftment model

All the animal studies were operated following a protocol approved by Institutional Animal Care & Utilization Committee of Chung Shan Medical University. For analysis of the expression of miR-494-3p to the tumorigenicity of BT-474 cells, cells were transduced with lentivirus carrying negative control or miR-494-3p precursor which were purchased from BioSettia (San Diego, CA, USA) for 3 days and injected into mammary fads of NOD/SCID mice (purchased from National Laboratory Animal Center, Taipei, Taiwan) as 1×10^6^ cells/50 μl Matrigel/site. For examine the *in vivo* therapeutic effect of hinokitiol, BT-474 cells were firstly cultured into secondary mammospheres and dissociated by HyQTase treatment. The dissociated secondary mammosphere cells were suspended in 2.5 mg/ml Matrigel (BD Biosciences) and injected into mammary fat pads as 2×10^4^ cells/50 μl/site. Hinokitiol treatment was performed intraperitoneally when tumors reached 100 mm^3^. Tumor volume was calculated as d^2^×D×π/6 where d and D were the shortest and longest diameter in mm, respectively [44].

### Immunohistochemistric analysis

Xenografted tumors were harvested, fixed with 3.7% formaldehyde and embedded into paraffin. 5 μm sections were sliced and the expression of BMI1 or ALDH1A1 was detected by polyclonal rabbit anti-BMI1 antibody (Novus Biologicals, LLC) or polyclonal rabbit anti-ALDH1A1 antibody (GeneTex Inc.) followed by a standard avidin-biotin-peroxidase complex method. 3,3'-Diaminobenzidine (DAKO, Carpinteria, CA) was then used to detect the antibody binding. The images of sections were scanned by TissueFAXS Plus (TissueGnostics GmbH, Vienna, Austria).

### Analysis of the association between miR-494-3p expression and overall survival rate in breast cancer patients

The association between miR-494-3p expression and overall survival rate of breast cancer patients was analyzed using public breast cancer datasets of GSE37405 and TCGA by online analysis tools. The dataset of GSE37405 was analyzed by MIRUMIR website (http://www.chemoprofiling.org/cgi-bin/GEO/MIRUMIR/web_run_MIRUMIR.V1.pl) whereas TCGA dataset was analyzed by PROGmiR V2 website (http://xvm145.jefferson.edu/progmir/).

### Statistical analysis

One-way analysis of variance (one-way ANOVA) was used to identify differences between experimental groups and the control group. A *P* value less than 0.05 was considered to be statistically significant.

## SUPPLEMENTARY MATERIALS FIGURE


