# Supplementary material for: Hinokitiol up-regulates miR-494-3p to suppress BMI1 expression and inhibits self-renewal of breast cancer stem/progenitor cells
Source: Oncotarget. 2017 Jun 27;8(44):76057–68. doi: 10.18632/oncotarget.18648 (PMC5652685; doi:10.18632/oncotarget.18648)
Supplement: Supplementary file 1 [file oncotarget-08-76057-s001.pdf]

# Hinokitiol up-regulates miR-494-3p to suppress BMI1 expression and inhibits self-renewal of breast cancer stem/progenitor cells

## SUPPLEMENTARY MATERIALS

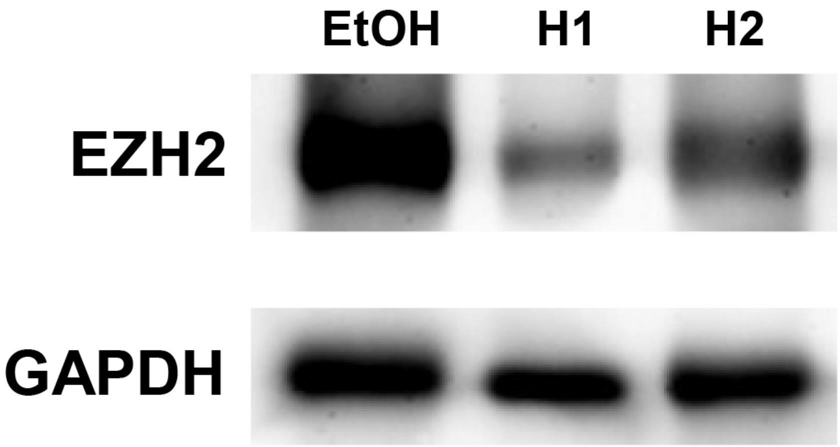


**Supplementary Figure 1: Hinokitiol inhibited EZH2 expression in BT-474 xenograft.** Total proteins were extracted from xenograft tumors of ethanol- (EtOH) or hinokitiol (H1 and H2)-treated mice and separated by SDS-PAGE. EZH2 expression was determined by western blot analysis. GAPDH was used as internal control.
